# Premenstrual Syndrome and the Attitudes Toward Seeking Professional Psychological Help Among College Students in Oman

**DOI:** 10.1089/whr.2024.0055

**Published:** 2024-09-30

**Authors:** Mohammed Qutishat, Lina Shakman, Safiya Alyaqoubi

**Affiliations:** ^1^ Community and Mental Health Department, College of Nursing, Sultan Qaboos University, Muscat, Sultanate of Oman.; ^2^ Child and Maternal Health Nursing Department, College of Nursing, Sultan Qaboos University, Muscat, Sultanate of Oman.

**Keywords:** premenstrual syndrome, seeking psychological help, college students

## Abstract

**Background:**

This study examines the relationship between premenstrual syndrome (PMS) and the attitude toward seeking professional psychological help among Omani college students.

**Method:**

This study used a cross-sectional design with a convenience sampling approach. The date was issued between January and March 2024. A sample of 601 undergraduate female students completed the study questionnaires, including (1) a sociodemographic questionnaire, (2) the Premenstrual Syndrome Scale and (3) the attitude toward seeking professional psychological distress scale.

**Results:**

The study included 601 eligible participants of age 18–29 years who were mostly single (83.7%) and living on campus (68.6%). PMS prevalence was high at 87.9%, with a mean score of 109.4, indicating low severity for most (62.1%). Participants generally had positive attitudes toward seeking psychological help (41.6% high willingness). A linear regression showed a positive link between PMS severity and help-seeking attitudes.

**Conclusion:**

This study highlights a significant association between the experiences of PMS and attitudes toward seeking professional psychological help among Omani female undergraduate students. With a high prevalence of PMS reported, the findings suggest that cultural factors and support systems play crucial roles in shaping these attitudes. The positive inclination toward seeking help indicates a growing awareness of mental health issues within this demographic. Enhancing mental health services and fostering supportive environments in educational settings can further empower students to address PMS-related challenges.

## Background

Premenstrual syndrome (PMS) is a health condition prevalent in 85%–90% of women of reproductive age,^1^ affecting the female mental, behavioral, and physical health conditions during the late luteal phase of their menstrual cycle.^2^ Symptoms typically last 6 days and disappear when menstruation begins, followed by a period of no symptoms.^3^ PMS prevalence is higher in unmarried women, women of age 35–44 years, and women from low socioeconomic groups living in socially deprived areas.^4^

The prevalence of PMS among college students of age 18–23 years ranges between 35% and 80%,^5^ possibly due to risk factors such as stress, age, marital status,^6^ maternal history,^7^ and ethnicity.^8^ Standard guidelines require a student to exhibit one emotional and somatic symptom in the preceding three menstrual cycles to be diagnosed with PMS.^9^ Common symptoms among college students include anxiety, distress, depression, exhaustion, irritability, skin conditions, swelling in the limbs, stomach bloating, backache, breast tenderness, decrease in appetite, and headaches.^9^

Premenstrual symptoms, especially in young students, can lead to challenges with academic performance, such as poor grades, absenteeism, dropping out of studies, attention problems, interpersonal relationships, mental health, and lower productivity.^10^ University students often lack the social and psychological tools to deal with daily life stresses, working hard to attain higher academic standing and achieve better professions.^11^

Efficiently managing PMS is crucial for enhancing female students’ academic performance and overall well-being.^12^ Those experiencing severe premenstrual symptoms may benefit from a combination of lifestyle changes and medical interventions, including regular exercise, stress reduction techniques such as meditation, and medications such as oral contraceptives or selective serotonin reuptake inhibitors (SSRIs).^13^ Previous research suggests that nonpharmacological approaches can effectively support PMS, with a comprehensive analysis of 32 studies showing significant symptom reductions without adverse effects, maintaining their benefits over time.^14^

PMS can be effectively treated with both cognitive behavioral therapy (CBT) and psychodynamic therapy.^15^ Cognitive behavioral therapy (CBT) aims to detect and change problematic thinking patterns and behaviors, as well as to provide coping skills for physical, emotional, and behavioral problems.^16^ In contrast, psychodynamic therapy investigates the underlying psychological and emotional reasons that may cause PMS, with the goal of resolving unresolved conflicts or traumas connected to the menstrual cycle in order to enhance emotional regulation and coping skills.^14^

In their responses to such symptoms, some students turn to self-treatment strategies to ease these symptoms, the most popular of which are analgesics such as painkillers, increasing the intake of hot liquids, massage, wearing warm clothing, lying prone, and altering one’s lifestyle,^17^ while most do not seek any treatment for their complaints.^18^ University students, however, may be reluctant to seek social or psychological support due to stigma. The willingness to seek psychological help depends on how its effects are perceived.^19^

The adoption of professional help-seeking behaviors for mental health issues among college students is currently gloomy and may decline in many countries.^20^ Factors influencing this include social stigma, lack of awareness about mental health, and barriers to accessing affordable and private services on campus.^21^ The unique developmental and social changes during college can also complicate seeking help. Attitudes toward mental health can indirectly affect students’ intentions to seek assistance, ultimately influencing their actions.^22,23^ This is supported by various studies in Arab countries examining student attitudes toward psychological help.^24–30^

This research focuses on the prevalence and impact of PMS among college students in Oman, emphasizing the importance of understanding cultural and social factors influencing their attitudes toward seeking psychological assistance. This study aims to identify gaps in health care access, raise mental health awareness, and promote a holistic approach to women’s well-being, providing insights for policies and interventions to improve support for this demographic. Despite the significance of these issues, there has been limited research on Omani students’ attitudes toward professional psychological treatment for PMS, making this study crucial for enhancing health care services and reducing the stigma surrounding mental health. For this reason, this study aimed to examine the prevalence of and relationship between PMS and the attitude toward seeking professional psychological help among Omani college students.

## Study Design

The Sultan Qaboos University College of Nursing’s Research Ethics Committee authorized the study’s conduct. A descriptive correlational study design was adopted to achieve the research’s aim among female undergraduate students. The research sample was chosen *via* convenience sampling. The researchers used power analysis to achieve a total sample of 450 college students given the following parameters: a 95% level of confidence and a 5% margin of error. Inclusion criteria included female students who had completed their foundation programs and bachelor’s degrees and were willing to participate in the study. It excluded, however, all students who had previous psychiatric problems, had irregular menstrual cycles, and were taking psychotropic medications.

The information center at the assigned university provided a list of female students’ email addresses, and the researchers sent invitation emails to all possible students on the mentioned list. The university provided the researchers with all email addresses that started with the student’s number (*e.g.,* S111XXX@squ.edu.om) to keep students’ information confidential. An online survey approach was used in the current investigation. The researchers created and presented the study questions using a Google form emailed to the students. Every student who took part completed written informed consent forms. Participants were informed that their participation in the survey was entirely voluntary and anonymous, and the study’s purpose, objective, methods, and potential benefits were discussed. All data were kept confidential, and only the research team could access it.

## Study Instruments

We used self-report instruments as a measurement tool to investigate the extent of the research phenomena. The instrument comprises three sections: (1) demographic data, (2) the Premenstrual Syndrome Scale (PMSS), and (3) the attitude toward seeking professional psychological distress scale. The PMSS and the attitude toward seeking professional psychological distress scale tools are available free online.^31,32^ The survey was given between January and March 2023 and it took an estimated 10–15 minutes to complete.

For accuracy, both tools were translated into Arabic and re-translated by bilingual and mental health experts in the College of Nursing and the Department of Maternal and Child Health. This includes translating the items from the original language (English) into Arabic, reviewing the Arabic translations to ensure accuracy, and then back-translating them into the original language. After that, the translation was compared with the originals, any discrepancies were identified, and the translation and back-translation process was repeated until the final versions accurately for both tools reflected the intended meaning and context.

### Premenstrual Syndrome Scale

The PMSS consists of 40 items divided into three subscales: physiological, psychological, and behavioral symptoms.^31,32^ This five-point Likert scale allows respondents to rate items using the following scoring system: “never” (1), “seldom” (2), “occasionally” (3), “frequently” (4), and “always” (5). Total scores range from 40 to 200, with a score of 80 or higher indicating the presence of PMS. Higher scores reflect greater severity of PMS symptoms. The level of PMS among the study participants was divided into three categories: high, medium, and low. This was adopted where scores exceeded the cutoff points of 80, calculated according to the formula (highest grade minus lowest grade, divided by three). Thus, the level of PMS was split into three levels: low (80–119 points), medium (120–159 points), and high (160–200 points). The scale has demonstrated strong test–retest correlation coefficients, temporal stability, and internal consistency in previous studies.^31,32^ In the current study, Cronbach’s alpha value was 0.946.

### Attitudes toward seeking professional psychological help scale

This scale measured participants’ attitudes toward seeking professional psychological help.^32^ It included two dimensions: openness to seeking professional help for emotional problems (five items), with item scores ranging from 0 (“disagree”) to 3 (“agree”), and the value of and need for seeking professional help (items 2, 4, 8, 9, and 10), with items scored in reverse. The total score on the scale ranges from 0 to 30, with higher scores indicating a better psychological help-seeking attitude. The cutoff score on the scale is greater than 20 points. The level of the attitude toward seeking psychological distress was also split into three levels. This was adopted where scores exceeded the cutoff points of 20, calculated according to a formula (highest grade minus lowest grade, divided by three). Based on that formula, help-seeking attitude was categorized as low (20–23.3), moderate (23.3–26.7), and high (26.7–30). The scale demonstrates the strength of test–retest correlation coefficients, temporal stability, and internal consistency.^32^ In the current study, Cronbach’s alpha value was 0.829.

## Data Analysis

The authors of this study used SPSS Statistics (version 24.0, Armonk, NY, USA) for statistical analysis. A *p*-value of <0.05 was considered significant. The results of this study were presented as means, standard deviations, frequencies, and percentages. Analysis of variance and a simple *t*-test were used to identify significant statistical differences between the study variables. Pearson’s correlation was used to detect the relationship between the attitude toward seeking psychological help and the experience of PMS among female undergraduate students in Oman. Linear regression was calculated to understand whether the experiences of PMS among female undergraduate students in Oman (the dependent variable) could be predicted by the attitude toward seeking professional psychological distress (the independent variable).

## Results

The researchers received 646 completed surveys. Afterward, the researchers attempted to make data clearance for all ineligible participants (6.96%, *n* = 45), including missing information (3.86%, *n* = 25), uncompleted questionnaires (4.64%, *n* = 30), and incorrect or ineligible participants (5.4%, *n* = 35). Ineligible participants include those with previous chronic medical or psychiatric health conditions (4.17%, *n* = 27). After data clearance, the investigator arrived at 601 samples. The participants ranged from 18 to 29 years old; most were single (83.7%, *n* = 503) and lived on campus (68.6%, *n* = 412). Around 39% of participants were in their second academic year (38.9%, *n* = 234) and scored B grades in the last academic semester (62.1%, *n* = 373). Table 1 details these results.

**Table 1.** Participant Demographics

Variable	Frequency	Percentage
Age		
18–20 years	83	13.8
21–23 years	382	63.6
24–26 years	132	22.0
27–29 years	4	0.7
Marital status		
Single	503	83.7
Married	98	16.3
Living arrangement		
On campus	412	68.6
Out campus	189	31.4
Academic year		
First year	96	16.0
Second year	234	38.9
Third year	166	27.6
Fourth year	71	11.8
Fifth year	34	5.7
GPA in the last semester		
A	120	20.0
B	373	62.1
C	103	17.1
D	5	0.8

### Level of PMS and attitudes toward seeking professional psychological help

The prevalence of PMS among the study participants was 87.85%. The mean score of the students’ experiences of PMS was 109.4 (SD = 26.1). The mean score of their attitudes toward seeking psychological help was 26.6 (SD = 5.7). The mean score for openness to seeking professional help for emotional problems was 13.9 (SD = 3.2), while the mean score for the value of and need for seeking professional help was 12.7 (SD = 3.2).

The result of this study shows that most of the study participants who exhibited PMS were at a low level (62.1%, *n* = 328) compared with the moderate (31.8%, *n* = 168) or high levels (6.1%, *n* = 32). On the other hand, most study participants demonstrated a high attitude toward seeking professional psychological help (41.6%, *n* = 227) compared with a moderate (36.1%, *n* = 197) or low level (22.2%, *n* = 121).

Table 2 summarizes student responses to the (PMSS, detailing the frequency of various symptoms. Notably, symptoms such as “weight gain” (44.9% sometimes) and “headache” (40.9% sometimes) are frequently reported, indicating significant concerns. “Anxiety” is particularly prominent, with 37.4% of respondents experiencing it “always.” Emotional symptoms such as “irritability” and “tension” also show high occurrences, suggesting a need for enhanced mental health support. Overall, the data highlight the varied impact of PMS on students, underlining the importance of targeted health resources.

**Table 2.** Student Responses to the Premenstrual Syndrome Scale

PMS symptoms	% (*n*)				
	Never	Rarely	Sometimes	Very often	Always
Breast tenderness	9.7 (58)	20.8 (125)	31.4 (189)	14.6 (88)	23.5 (141)
Abdominal bloating	6.3 (38)	24.6 (148)	35.6 (214)	3.8 (23)	29.6 (178)
Weight gain	9.8 (59)	27.5 (165)	44.9 (270)	1.8 (11)	16 (96)
Headache	8.2 (49)	30.9 (186)	40.9 (246)	2.8 (17)	17.1 (103)
Dizziness	17.0 (102)	30.3 (182)	37.4 (225)	2.2 (13)	13.1 (79)
Fatigue	9.5 (57)	28.3 (170)	38.9 (234)	4.3 (26)	19.0 (114)
Palpitation	12.6 (76)	30.8 (185)	38.9 (234)	2.8 (17)	14.8 (89)
Pelvic pain	9.7 (58)	28.5 (171)	34.1 (205)	6.3 (38)	21.5 (129)
Cramps	10.3 (62)	29.8 (179)	32.4 (195)	6.7 (40)	20.8 (125)
Change bowel habit	13.0 (78)	31.3 (188)	30.4 (183)	6.5 (39)	18.8 (113)
Increase appetite	13.3 (80)	32.9 (198)	31.3 (188)	6.0 (36)	16.5 (99)
Generalized aches	11.8 (71)	33.3 (200)	29.1 (175)	5.8 (35)	20.0 (120)
Food craving	13.0 (78)	31.1 (187)	31.1 (187)	7.5 (45)	17.3 (104)
Skin changes	17.3 (104)	28.3 (170)	33.1 (199)	5.7 (34)	15.6 (94)
Nausea and vomiting	18.1 (109)	28.6 (172)	32.1 (193)	5.2 (31)	16.0 (96)
Muscle pain	13.1 (79)	26.8 (161)	32.3 (194)	8.3 (50)	19.5 (117)
Irritability	8.2 (49)	13.1 (79)	24.8 (149)	26.5 (159)	27.5 (165)
Anxiety	6.3 (38)	19.3 (116)	33.8 (203)	3.2 (19)	37.4 (225)
Tension	5.8 (35)	20.1 (121)	50.1 (301)	3.5 (21)	20.5 (123)
Mood swings	5.2 (310)	27.8 (167)	37.6 (226)	6.7 (40)	22.8 (137)
Loss of concentration	11.5 (69)	27.1 (163)	36.6 (220)	4.5 (27)	20.3 (122)
Depression	11.0 (66)	30.4 (183)	35.4 (213)	4.5 (27)	18.6 (112)
Forgetfulness	13.3 (80)	32.3 (194)	33.8 (203)	2.7 (16)	18.0 (108)
Crying	11.5 (69)	28.3 (170)	34.8 (209)	6.5 (39)	19.0 (114)
Sleep changes	10.5 (63)	31.3 (188)	33.9 (204)	5.0 (30)	19.3 (116)
Being over-sensitive	11.1 (67)	27.8 (167)	34.1 (205)	8.8 (53)	18.1 (109)
Irrational thought	14.1 (85)	30.4 (183)	37.3 (224)	4.7 (28)	13.5 (81)
Compulsive behaviors	14.3 (86)	34.8 (209)	32.9 (198)	4.0 (24)	14.0 (84)
Obsessional thought	11.8 (71)	34.1 (205)	34.4 (207)	6.5 (39)	13.1 (79)
Impaired work performance	13.0 (78)	32.8 (197)	35.9 (216)	3.7 (22)	14.6 (88)
Poor judgment	12.1 (73)	32.1 (193)	36.3 (218)	3.2 (19)	16.3 (98)
Lack of interest	9.5 (57)	29.5 (177)	36.4 (219)	5.3 (32)	19.3 (116)
Clumsiness	11.6 (70)	26.8 (161)	36.3 (218)	4.8 (29)	20.5 (123)
Feeling guilty	6.7 (40)	30.9 (186)	39.6 (238)	3.8 (23)	19.0 (114)
Aggression	11.5 (69)	30.9 (186)	30.8 (185)	7.8 (47)	19.0 (114)
Confusion	10.6 (64)	35.4 (213)	30.6 (184)	5.3 (32)	18.0 (108)
Hopelessness	13.0 (78)	29.0 (174)	32.6 (196)	8.2 (49)	17.3 (104)
Lack of self-control	5.0 (30)	21.6 (130)	50.6 (304)	3.8 (23)	19.0 (114)
Restlessness	5.3 (32)	21.6 (130)	30.4 (183)	4.2 (25)	38.4 (231)
Social withdrawal	5.7 (34)	15.6 (94)	26.5 (159)	29.1 (175)	23.1 (139)

The results of this study indicate a significant difference in experiences of PMS among the study participants’ marital status (*t* = 3.4, *p* < 0.001). On the other hand, the results of the current study found no significant differences in overall PMS experiences and the attitude toward seeking professional psychological help across the other demographic data. Table 3 details these results.

**Table 3.** Descriptive Statistics of Premenstrual Syndrome Experiences and the Attitude Toward Seeking Professional Psychological Help

Variable	Attitude toward seeking help				Premenstrual syndrome			
	Mean	SD	Test*	*p*-Value	Mean (SD)	SD	Test*	*p*-Value
Age								
18–20 years	26.5	5.5	*F* = 0.4	0.8	114.7	30.1	*F* = 2.6	0.05
21–23 years	26.5	5.5			109.7	24.9		
24–26 years	26.9	6.4			104.0	26.6		
27–29 years	24.7	2.5			116.0	41.6		
Marital status								
Single	26.6	5.4	*t* = 0.4	0.7	110.9	25.7	*t* = 3.4	*p* < 0.001
Married	26.3	6.6			101.2	26.8		
Living arrangement								
On campus	26.1	5.3	*t* = 1.2	0.2	111.3	25.5	*t* = 1.2	0.2
Out campus	26.8	5.8			108.4	26.3		
Academic year								
First year	26.2	5.0	*F* = 0.754	0.6	112.9	26.7	*F* = 0.9	0.5
Second year	26.9	6.0			107.7	26.7		
Third year	26.8	6.0			107.8	24.4		
Fourth year	25.7	5.1			111.2	26.4		
Fifth year	25.9	4.1			113.9	27.6		
GPA in the last semester								
A	26.3	5.7	*F* = 0.254	0.8	112.3	26.3	*F* = 0.8	0.5
B	26.6	5.5			108.9	25.7		
C	26.7	6.2			107.5	26.6		
D	28.4	6.9			113.8	41.6		

*Significant at 0.05.

### Relationship between level of PMS and attitudes toward seeking professional psychological help

To understand further whether the experiences of PMS (dependent variable) among undergraduate students in Oman could be predicted by their attitude toward seeking psychological help (independent variable), a linear regression was calculated, and after adjusting for the effect of confounding variables, the results indicate that a higher degree of PMS shown to be positively and significantly associated with a positive attitude toward seeking psychological help (*F* = 74.9, *p* < 0.001). The results showed that PMS explained 11% of the variation in attitude toward seeking psychological help. Table 4 details these results.

**Table 4.** Results of Linear Regression

Predictor	Unstandardized coefficients		Standardized coefficients	t	Sig.	95% confidence interval for *B*	
	B	Standard error	Beta			Lower bound	Upper bound
Attitude toward seeking professional psychological help	1.528	0.18	0.33	8.7	0.000	1.18	1.88

## Discussion

The current study investigated the association between the experiences of PMS and the attitude toward seeking professional psychological help among female undergraduate students in Oman. This study found that the mean scores of the experiences of PMS and the attitude toward seeking professional psychological help were 109.4 (SD = 26.1) and 26.6 (SD = 5.7), respectively. It also indicated that the prevalence of PMS among the study participants was 87.9%. Most were at the lower experience level of PMS (62.1%, *n* = 328) compared with the moderate and high levels found in other previous studies.^33^ The prevalence of PMS among students in our study, however, was much higher than that found in other studies reported in Taiwan and Thailand (28–39.5%),^34–36^ The United Arab Emirates (35%),^37^ Egypt (86.3%),^38^ and Saudi Arabia (80.1%).^39^ Nevertheless, this prevalence is lower than that found in Jordan (91%)^40^ and Bahrain (96%).^41^

The rate variation is likely linked to the difference in sample types used in this study compared with the targeted samples in other studies. While previous studies included females from various age groups, including college students, this study focused on university-aged females. Consequently, the factors such as the types and intensity of stressors, cultural outlook on females’ roles and responsibilities during their college years, and sociocultural influences that significantly contribute to the development of these symptoms may vary.^42,43^

Additionally, the stressors and lifestyle changes associated with college life, such as academic obligations, extracurricular activities, social pressures, and abnormal sleep habits, can significantly worsen PMS symptoms.^44^ Furthermore, college students’ dietary and exercise habits frequently differ from the general population, with characteristics such as irregular meals, high consumption of processed foods, caffeine, and sedentary activity all possibly contributing to the exacerbation of PMS.^45^

The academic environment in the college system may impose enormous expectations on students, asking them to balance a significant course load, tight study schedules, and frequent exams or evaluations.^46,47^ This tremendous academic pressure and the desire to maintain excellent grades and performance can result in increased stress, worry, and emotional strain, all of which can worsen PMS symptoms.^11^

Despite the high frequency of PMS, Omani college students may have fewer symptoms for various cultural and societal reasons. In Oman, specific cultural ideas, attitudes, or behaviors related to menstruation and women’s health may affect how college students perceive and report premenstrual symptoms.^48^ These cultural influences may influence how people understand and cope with the physical and emotional changes caused by PMS.^49^

Furthermore, Omani college students may have unique coping skills or support networks in place that help reduce the effect of premenstrual symptoms, resulting in a lower reported degree of severity, which is highly supported by many previous studies in different countries.^50^ Such strategies identified in previous studies are active planning, using humor, emotional support, and self-distractions.^51^ Another reason is that Omani college students have access to health care facilities such as student clinic, students’ council center, and student affairs center, and their understanding of PMS may differ from other demographics, affecting symptom detection and management. Individual differences in symptom perception and reporting, as well as hereditary or hormonal variables, may help explain the disparity between the high frequency of PMS and the lower levels of its reported symptoms among Omani college students.

The study results revealed significant variations in PMS experiences based on the participants’ marital status. It was observed that single students had significantly higher levels of PMS compared with married individuals (*p* < 0.001). The average PMS scores for single and married students were 111 (SD = 25.7) and 101.1 (SD = 26.8), respectively. This difference could be attributed to the fact that married individuals typically benefit from more stable relationships and emotional support from their partners, which may help alleviate the effect of PMS symptoms.^52^ Furthermore, the hormonal and physiological alterations that take place due to marriage and intimate relationships may also have an impact on regulating PMS encounters.^53^ Possible explanations for the variations in PMS among single and married Omani students may include alterations in menstrual cycle patterns, ovulation, and levels of reproductive hormones.^54^ Moreover, the sociocultural environment in Oman, characterized by distinct customs and societal expectations on marriage, can influence how Omani women perceive and feel PMS, depending on their marital status. The societal stigma and social constraints linked to singleness in Omani society have the potential to worsen PMS among unmarried students.^55^

Most study participants demonstrated a high attitude toward seeking professional psychological help (41.5%, *n* = 227) compared with other previous studies.^26,56^ Several variables may contribute to Omani college students’ willingness to seek psychological professional treatment. First, there has been a rise in national awareness of mental health concerns, resulting in a lower stigma associated with getting care for psychological difficulties.^57^ Second, the current study participants were more inclined to seek professional psychological help than previous research on college students in Oman. This difference can primarily be attributed to the timing of the studies and the changing societal landscape concerning mental health.^58^ The present study documented a time of increased receptiveness and tolerance among university students, mirroring a more extensive change in Oman toward emphasizing psychological well-being. This shift may be attributed to heightened mental health awareness campaigns, enhanced availability of mental health services, modifications in educational curricula and campus culture, and a general transformation in societal attitudes and norms regarding the significance of seeking professional psychological assistance.^59^ By using this more advantageous environment, the present study revealed a greater prevalence of positive attitudes toward seeking assistance among the college student demographic.

Furthermore, schools and universities in Oman may offer a friendly environment that encourages students to prioritize their mental health and seek professional treatment when necessary.^60^ There may also have been a societal change toward acknowledging the value of mental well-being, resulting in a greater acceptability of obtaining psychiatric care among college students. The availability of psychiatric services on college campuses or in the community may also influence students’ decisions to seek professional treatment.^61^

The results indicate that a higher degree of PMS is positively and significantly associated with a positive attitude toward seeking psychological help (*p* < 0.001). The reasons for this are, first, that individuals with a greater level of PMS may notice that the severity of their symptoms has a detrimental influence on their everyday and emotional well-being. As a result, college students may be more likely to seek psychological therapy as a proactive measure for controlling their symptoms and increasing their quality of life.^30^ The cyclical pattern of symptoms might lead people to seek psychological therapy as a coping method for adequately managing their symptoms. PMS symptoms might worsen underlying psychological disorders or necessitate further care.^62^ Severe PMS symptoms may indicate the need for psychological therapy to manage emotional issues throughout one’s menstrual cycle.^63^

Additionally, cultural variables in Oman may affect undergraduate students’ views about obtaining psychological treatment.^47^ The stigma connected with mental health concerns may make people hesitant to seek professional help.^30^ The experience of PMS, however, might act as a springboard for people to transcend this stigma and seek care for their mental health. Individuals with severe PMS symptoms who seek psychological therapy may benefit from therapeutic approaches, coping skills, and support systems that can help them better navigate the challenges of PMS.

The correlation between increased severity of PMS and a more positive inclination toward seeking psychological assistance among college students in Oman is likely to be significantly impacted by the influence of peers and the level of social support within the campus setting.^64^ The cohesive college community can influence individual perspectives, as favorable accounts and collective encounters of fellow students who have sought assistance for PMS-related concerns can standardize and remove the stigma associated with obtaining treatment.^65^ Engaging in open dialogues regarding the difficulties associated with managing PMS symptoms alongside student organizations or campus initiatives that advocate for mental health awareness and provide access to counseling services can foster a culture of understanding and validation.^66^ This culture can motivate women to prioritize their well-being and seek professional assistance. In addition, if college administrators and faculty members exhibit comprehension and actively advocate for the utilization of mental health resources, it can further strengthen the notion that seeking assistance for PMS is a legitimate and encouraged measure, empowering college students in Oman to address their premenstrual challenges through psychological support.^67,68^

### Study Implications

Examining the correlation between PMS and perspectives on obtaining professional psychological assistance among university students in Oman has noteworthy consequences for several aspects of personal and community welfare. This study contributes to better knowledge of the effects of PMS on mental health, which will raise awareness of the significance of treating PMS-related mental health problems among college students.

Additionally, by examining how PMS affects attitudes toward seeking professional psychological assistance, researchers can lessen the stigma associated with discussing women’s health and mental well-being and create a more accepting and supportive environment for those dealing with PMS symptoms.^40^

Second, it is imperative to enhance the availability of mental health services to enable college students to obtain expert psychological support. The number of mental health practitioners and financing for such services may be increased by policymakers and university administrators, particularly those with prior expertise in the medical field.^33^ Telehealth services can increase accessibility by offering discreet and convenient assistance.^69^ Students can receive appropriate care while experiencing less stress and worry thanks to telemedicine facilities and platforms.^23^

Third, legislators must consider offering peer-support groups, private counseling services, and other specialized support systems for college students. Through these platforms, students can talk to qualified peers about their mental health concerns and receive emotional support.^70^ Since these systems create a safe space in which students feel comfortable disclosing their struggles, they can enhance the availability and effectiveness of psychological treatment among them. This can enhance their well-being and quality of learning and lessen the stigma associated with attending therapy.^71^ Peers with comparable experiences can offer empathetic support, normalizing the need for help and providing consolation. Doing so builds confidence among colleagues and ensures that assistance is successful.^72^

Overall, researchers can create a more thorough strategy for improving mental health and fostering holistic well-being among those affected by PMS in educational settings by exploring the connection between PMS and attitudes toward seeking professional psychological help among college students in Oman.

### Limitations

The study’s limitations include its online structure and the need to monitor the students’ long-term mental- and physical-health histories. These limits highlight the need to carefully analyze the results and identify areas for improvement in future studies to improve the accuracy and usefulness of the outcomes in the Omani context.

Another limitation of this study is the predominant focus on the psychological aspects of PMS, which may overshadow other treatment options such as medical and nonpharmacological approaches, potentially influencing students’ attitudes toward seeking help. Furthermore, the lack of inquiry into whether participants were presented with alternatives to psychological help when discussing PMS symptoms restricts the comprehensiveness of the findings and warrants further exploration in the Discussion section above.

Another inherent constraint of the present study is the absence of sociodemographic variables, such as age or years of study, in the regression analysis. In order to enhance the informativeness and validity of the regression model, it is advisable to incorporate these specific determinants. By including variables, such as age and years of study, the analysis could offer a more comprehensive comprehension of the elements that influence the desired outcome.

Future research in this field should utilize a multinomial logistic regression method that incorporates pertinent sociodemographic factors, such as age and years of study, as predictors in the model. Conducting this form of analysis would enable a more comprehensive investigation of the relationships between these factors and the result variable.

## Conclusion

The study investigates the correlation between PMS experiences and the desire to seek professional psychological help among female undergraduate students in Oman. The results show a high prevalence of PMS, with most experiencing lower levels of PMS. The study suggests that cultural norms and support systems may mitigate symptom severity, while marital status can also affect the severity of PMS. Single students reported higher scores than married individuals, possibly due to the emotional support they receive from stable relationships. The study also found a strong positive association between higher PMS severity and a favorable attitude toward seeking psychological help. The study suggests that cultural factors and evolving societal attitudes in Oman may influence students’ perceptions of mental health care, leading to an increased willingness to seek psychological support. The study emphasizes the importance of addressing PMS experiences and promoting mental health awareness among college students. Encouraging a proactive approach to seeking psychological help can improve coping strategies and support systems, ultimately fostering better management of premenstrual symptoms and overall well-being.

## Abbreviations Used

CBT: Cognitive Behavior Therapy
PMS: Premenstrual syndrome
PMSS: Premenstrual Syndrome Scale
SSRI: Selective Serotonin Reuptake Inhibitor
